# Epoxy-Functionalized Isatin Derivative: Synthesis, Computational Evaluation, and Antibacterial Analysis

**DOI:** 10.3390/antibiotics14060595

**Published:** 2025-06-09

**Authors:** Deepanjali Shukla, Iqbal Azad, Mohd Arsh Khan, Ziaul Husain, Azhar Kamal, Sabahat Yasmeen Sheikh, Ibrahim Alotibi, Varish Ahmad, Firoj Hassan

**Affiliations:** 1 Department of Chemistry, Integral University, Lucknow 226026, India; djshukla@student.iul.ac.in (D.S.); arshkn@student.iul.ac.in (M.A.K.); ziaulh@student.iul.ac.in (Z.H.); sabahats@iul.ac.in (S.Y.S.); 2 Department of Bioengineering, Integral University, Lucknow 226026, India; azharbio@iul.ac.in; 3 Health Information Technology Department, The Applied College, King Abdulaziz University, Jeddah 21589, Saudi Arabiavaahmad@kau.edu.sa (V.A.)

**Keywords:** ADMET, antibacterial, dam protein, epoxy-functionalized isatin, *K. pneumoniae*, molecular docking

## Abstract

Background/Objectives: The current need for new antibacterial compounds that target non-classical pathways is highlighted by the emergence of multidrug-resistant *Klebsiella pneumoniae*. In the development of antibiotics, DNA adenine methyltransferase (Dam), a key regulator of bacterial gene expression and pathogenicity, is still underutilized. Epoxy-functionalized analogues of isatin derivatives have not been adequately investigated for their antibacterial activity, particularly as Dam inhibitors. In the pursuit of antimicrobial agents, this study synthesized an epoxy-functionalized isatin derivative (**L3**) using a one-pot reaction. The compound was characterized using FT-IR, ¹H-NMR, ^13^C-NMR, HR-MS, and UV–Vis spectroscopy. Methods: *In silico* evaluation performed by using ADMETlab3 and SwissADME. While molecular docking studies were achieved by AutoDock and Vina to find **L3**’s interaction with potential antibacterial target (Dam protein in *K. pneumoniae*). In addition, the antibacterial potential of **L3** was evaluated using minimum inhibitory concentration (MIC) assays against *Bacillus cereus*, *Bacillus pumilus*, *Escherichia coli*, and *K. pneumoniae*. Results: Among these, **L3** exhibited potential inhibitory activity against *K. pneumoniae*, with a MIC value of 93.75 μg/mL. *In silico* evaluations confirmed **L3**’s favorable drug-like properties, including potential oral bioavailability, blood–brain barrier (BBB) permeability, and low plasma protein binding (PPB). The compound satisfied Lipinski’s and other drug-likeness rules as well as getting a quantitative estimate of drug-likeness (QED) score of 0.52. Here, a homology model of Dam protein in *K. pneumoniae* was generated using the SWISS-MODEL server and validated using computational tools. Targeted docking analysis revealed that **L3** exhibited significant potential binding affinity against Dam protein, with binding energies of −6.4 kcal/mol and −4.85 kcal/mol, as determined by Vina and AutoDock, respectively. The associated inhibition constant was calculated as 280.35 µM. Further interaction analysis identified the formation of hydrogen bonds with TRP7 and PHE32, along with Van der Waals’ interactions involving GLY9, ASP51, and ASP179. Conclusions: These findings highlight **L3** as a promising scaffold for antimicrobial drug development, particularly in targeting Dam protein in *K. pneumoniae*. Furthermore, the ADMET profiling and physicochemical properties of **L3** support its potential as a drug-like candidate.

## 1. Introduction

The worldwide rise in multidrug-resistant (MDR) bacterial infections possess a severe threat to public health and calls for the quick development of new antimicrobial drugs, particularly those produced by *Klebsiella pneumoniae* [1,2,3]. Traditional antibiotics are increasingly ineffective due to diverse resistance mechanisms. Consequently, innovative therapeutic approaches targeting unidentified microbial pathways are in high demand [4,5,6].

In view of this, isatin (1H-indole-2,3-dione), a heterocyclic compound of significant pharmacological interest [7,8], has received attention because of its privileged scaffold and wide range of biological activities [9,10]. Structurally adaptable, isatin functions as a useful synthon in the field of drug design and development (DDD), especially when modified at the nitrogen atom [11,12,13,14]. Among the different *N*-substituted isatin derivatives, reactive functional groups like epoxides show promising medicinal potential due to their capacity to establish covalent interactions with biological targets [15,16,17,18,19]. The incorporation of an epoxy group into the *N*-substituted isatin structure offers a strategic method in medicinal chemistry [20,21,22]. Epoxides, known for their strained three-membered ring, are electrophilic and can be subjected to nucleophilic attack by amino acid residues found in enzymes and proteins [23,24,25]. This reactivity increases the likelihood of these compounds serving as irreversible enzyme inhibitors or as modulators of biological pathways [26,27]. As a result, isatin derivatives that contain an epoxy group are being investigated more frequently for their antimicrobial, anticancer, antiviral, and anti-inflammatory effects [8,28,29]. The combination of the isatin core with an oxirane (epoxide) group not only expands the chemical diversity but also allows for the fine-tuning of physicochemical and pharmacokinetic characteristics [21,30,31].

In 2013, Nisha et al. reported β-amino alcohol-based β-lactam–isatin chimeras from a 3-amino-2-azetidinone and 1-oxiranylmethyl isatin (**L3**) intermediate with the help of an acid-catalyzed epoxide ring opening mechanism. Preliminary base-promoted allylation of C-5-substituted isatins yields *N*-allyl isatin, which underwent epoxidation using m-chloroperbenzoic acid (mCPBA) in dry chloroform at 60°C to synthesize the intermediate **L3** [32]. Similarly, in 2014, Nisha et al. again successfully reported β-amino-alcohol-tethered 4-aminoquinoline-isatin conjugates from 4-aminoquinolines and 1-oxiranylmethyl isatin (**L3**) intermediates. In this study, intermediate **L3** was successfully prepared using the previously mentioned reaction condition [33]. Recently, Li et al. (2022) successfully reported the synthesis of novel metronidazole-derived three-component hybrids using the 1-(oxiran-2-ylmethyl) indoline-2,3-dione intermediate (**L3**) from 2,3-indolinedione and 2-(chloromethyl) oxirane under the reaction condition of 12 h and 60 °C in the presence of anhydrous K_2_CO_3_ and CH_3_CN [34]. Similarly, Li et al. (2022) also successfully reported phenylhydrazone-based oxindole-thiolazoles using 2-(chloromethyl)-oxirane and oxindole (**L3**) under the same reaction condition [35].

**Figure 1:**
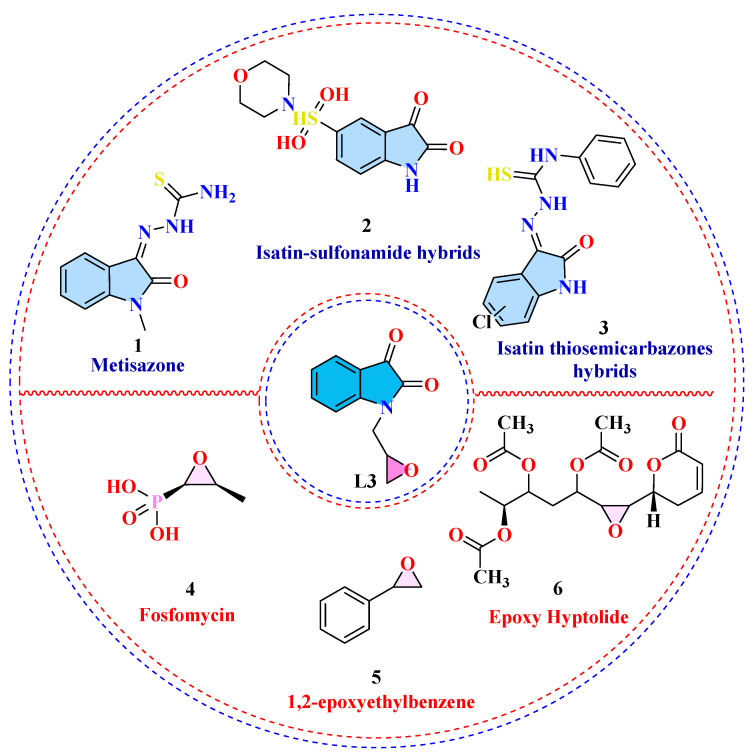
Structures of metisazone (1), isatin-sulfonamide hybrids (2), isatin thiosemicarbazone hybrids (3), fosfomycin (4), 1,2-epoxyethylbenzene (5), epoxy hyptolide (6), and the epoxy-functionalized isatin derivative (**L3**).

Therefore, the isatin core with oxygen produced different hybrids with various biological activities. Thus, it exhibits multi-target-directed ligand behavior, a beneficial characteristic for managing intricate conditions such as cancer and infectious diseases [36,37,38]. According to the literature survey, some of the reported compounds with isatin moieties contain antimicrobial properties; for example, metisazone (1) [39,40]. Isatin-sulfonamide hybrids (2) are antibacterial agents against Gram-positive (*Staphylococcus aureus*, *Staphylococcus epidermidis*, and *Bacillus subtilis*) bacteria and Gram-negative (*Proteus vulgaris*, *Klebsiella pneumoniae*, and *Shigella flexneri*) bacteria with an MIC of 0.007–0.49 µg/mL [9,41]. Isatin thiosemicarbazone hybrids (3) are active with a minimum inhibitory concentration (MIC) value of approx. 1.56 mg/L against Gram-positive bacterial strains: *Staphylococcus aureus* (ATCC 6538) and *Bacillus subtilis* (ATCC 6633) [42]. On the other hand, few compounds possess an epoxide ring and exhibit various biological activities. For example, fosfomycin (4) is a broad-spectrum bactericidal antibiotic produced by certain *Streptomyces* species that compromises cell wall synthesis in Gram-positive and Gram-negative bacteria by inhibiting the first step involving phosphoenolpyruvate synthetase. At present, it is used to treat urinary tract infections among other kinds of infections [43,44]. In addition, 1,2-epoxyethylbenzene (5) (at its sublethal MIC:13 µM) potentially inhibits gelatinase output controlled by the *fsr* QS system. However, the IC_50_ dose (6 µM) of 1,2-epoxyethylbenzene (5) only slightly reduced gelatinase production in *E. faecalis* OU510 under a concentration (10 nM) of GBAP, suggesting that 1,2-epoxyethylbenzene (5) influences GBAP signal transduction more than GBAP biosynthesis [45]. It has been noted that the presence of an epoxy ring enhances antibacterial activity. The *Hyptis pectinata* extract, hyptolide, and epoxy hyptolide (6) were evaluated against Gram-negative bacteria. *Escherichia coli* and *Salmonella thyposa* were included in the evaluation. The antibacterial activity against Gram-negative *Salmonella thyposa* (21.80 ± 0.74 IZ) is more effective than *Escherichia coli* (14.00 ± 0.42 IZ) due to the high concentration of epoxy hyptolide (6) present [46,47] (Figure 1).

Although isatin derivatives have shown a broad spectrum of biological activity, the incorporation of reactive functional groups, such as epoxides, into isatin scaffolds is still open for antimicrobial studies. Due to its role in regulating bacterial genes and virulence, DNA adenine methyltransferase (Dam) has not been well investigated in medicine development pathways. Consequently, there is a current need to develop isatin-based compounds with the potential antibacterial activity and favorable therapeutic properties able to effectively target Dam protein. The lack of experimental evidence supporting the mechanism of action, toxicity profile, and pharmacological scope of these compounds. Nevertheless, it presents a serious flaw in the process of converting these leads into potential therapeutic candidates. Thus, numerous studies have highlighted the importance of *N*-substitution on isatin in influencing biological activity, where a minor alteration in substitution patterns can result in considerable variations in activity profiles [48,49,50,51,52,53,54,55]. The existence of an epoxy ring further increases the reactivity and probable biological activity contribution, making such molecules appealing candidates for lead optimization during DDD [56,57].

In this regard, the present study centers on the synthesis, characterization, *in silico* ADMET analysis, and biological assessment of an epoxy-functionalized isatin derivative (1-oxiranylmethyl isatin/1-(oxiran-2-ylmethyl)indoline-2,3-dione/oxindole/**L3**) that includes an epoxy group. This study aims to investigate the compound’s antibacterial properties and evaluate its drug-likeness and pharmacokinetic characteristics, thereby aiding the development of potential therapeutic agents based on the isatin scaffold.

## 2. Results

### 2.1. Chemistry

**Scheme 1:**
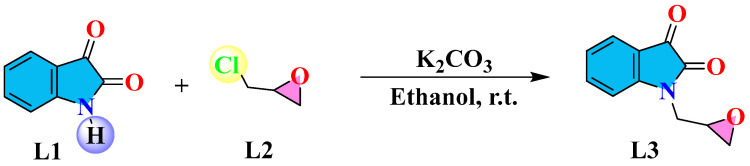
Synthesis of epoxy-functionalized isatin derivative (**L3**).

In this study, a one-pot reaction was used to prepare **L3**. A stirred mixture of isatin (**L1**) (1 mmol, 0.147g) and epichlorohydrin (**L2**) (1 mmol, 77.80 μmL) was allowed to react together in the presence of ethanol (6 mL) under a mild condition including anhydrous potassium carbonate (0.5 mmol, 0.069 g) at room temperature for about 3–4 h. The progress of the reaction was monitored with the help of thin-layer chromatography (TLC) (n-hexane:ethyl acetate = 7:3). After completion of the reaction, the solvent was removed under reduced pressure. Brown color powdered precipitate was formed, washed with ethanol, filtered, and dried at room temperature. The powdered precipitate of the residue was collected. Then, the residue obtained was purified using column chromatography, resulting in the desired product **L3** as an orange solid (2.826 g, 13.91 mmol) with a yield of 78% (Scheme 1) [34,35].

**Figure 2:**
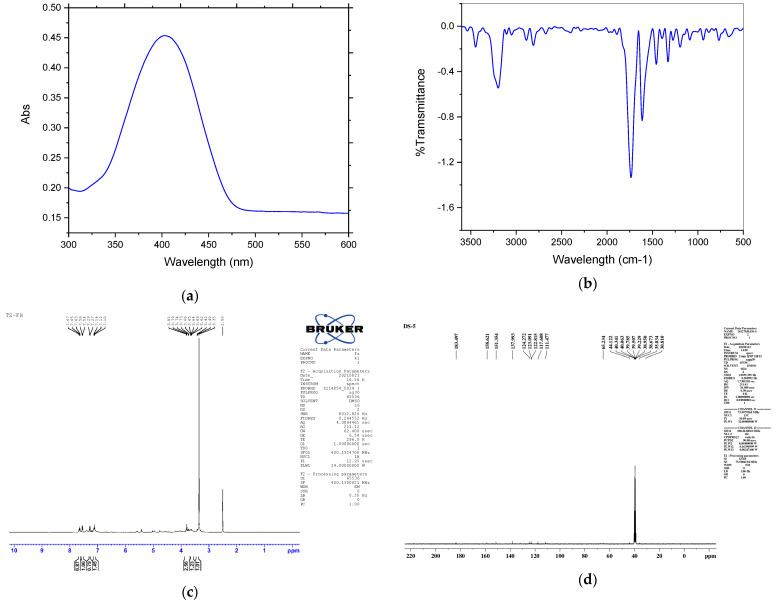
The characterization details of the epoxy-functionalized isatin derivative (**L3**). (**a**) UV–Vis plot; (**b**) FT–IR plot; (**c**) ^1^H–NMR, and (**d**) ^13^C–NMR spectra.

In this study, FT-IR, ^1^H-NMR, ^13^C-NMR, HR-MS, and UV–Vis were used to determine the structure of synthesized compound (**L3**) (Figure 2).

### 2.2. Characterization Details

#### Epoxy-Functionalized Isatin Derivative (1-(Oxiran-2-ylmethyl)indoline-2,3-dione; **L3**)

Orange solid, yield: 78%; UV–Vis (H_2_O): λ_max_ (nm) = 403 (A = 1.2); FT-IR ((KBr) νmax (cm^−1^)): 880 (C-H, epoxide), 1194 (C-O-C, epoxy), 1328 (C-N, from epoxide), 1614 (C=O, isatin), 1727 (C=O, isatin), 2812, 2888 (aliphatic N-CH_2_), 3051, 3106, 3191, (aromatic CH); ^1^H NMR (400 MHz, DMSO-d_6_, ppm) δ_H_: 7.67–7.63 (1H, t, CH-Ar, J = 7.6 Hz), 7.56–7.54 (1H, d, CH-Ar, J = 7.5 Hz), 7.29–7.27 (1H, d, CH-Ar, J = 7.3 Hz), 7.14–7.10 (1H, t, CH-Ar, J = 7.1 Hz), 3.81–3.73 (3H, m), 3.66–3.62 (1H, m, CH-epoxy), 3.42–40 (1H, d, CH_2_-epoxy, J = 3.4 Hz); ^13^C-NMR (75 MHz, DMSO-d_6_) δ_c_; 30.8, 65.2, 111.4, 117.6, 122.8, 123.0, 124.2, 137.9, 151.3, 158.6, 183.4; HR-MS m/z (C_11_H_9_NO_3_): calcd., 203.06; found: 204.06 [M + H]^+^.

### 2.3. In Silico Evaluation of Drug-Likeness and ADMET Parameters

#### 2.3.1. Physiological Properties

The epoxy-functionalized isatin derivative (**L3**) may exhibit characteristics associated with drug-likeness. The molecular weight of the synthesized compound is 203.19 g/mol, indicates its favorable oral bioavailability. Four heteroatoms among the 15 total atoms generate moderate polarity. Its relatively stiff structure, three-ring system, and only two rotatable bonds suggest a rigid configuration that favors membrane permeability and target specificity. Furthermore, its potential to cross biological membranes is enhanced by absence of hydrogen bond donors (HBD = 0) and the presence of just three hydrogen bond acceptors (HBA = 3). Its topological polar surface area (TPSA) is 49.91 Å^2^, which supports potential permeability and shows its possibility to cross the blood–brain barrier (BBB). With a slightly hydrophilic character that strikes a potential comparison between lipophilicity and aqueous solubility, both of which are essential for absorption and distribution, the compound’s partition coefficient (logP) is 0.615. These properties indicate that **L3** probably is appropriately absorbed orally and displays a moderate to good distribution with a chance to reach central nervous system (CNS) targets (Table 1). Its small size and moderate polarity also point to primarily kidney excretion or metabolism *via* Phase II conjugation pathways. It’s encouraging physiological properties present **L3** fit for additional development as a bioactive molecule.

A quantitative estimate of drug-likeness (QED) score of 0.52 suggests that **L3** has a balanced set of physicochemical properties suitable for oral medications, but there is still scope for potency or selectivity optimization. Compound **L3** meets Lipinski’s rule of five (MW < 500, logP < 5, HBD ≤ 5, HBA ≤ 10), which suggests potential oral bioavailability because its molecular weight, lipophilicity (logP), hydrogen bond donors, and acceptors values are reasonable. Furthermore, it passes the Pfizer rule showing low chances of cardiotoxicity as well as other toxicity hazards usually related to reactive features or high lipophilicity. **L3** also satisfies the GSK rule known as the 4/400 rule, which identifies compounds with a high chance of poor solubility or metabolic instability. **L3** has a low SlogP of 0.615 and a molecular weight of 203.197; thus, it is well below the thresholds showing potential developability and lower probability of attrition during pharmaceutical development (Table 2).

#### 2.3.2. ADMET Properties

The epoxy-functionalized isatin derivative (**L3**) demonstrates favorable pharmacokinetic and toxicity characteristics, indicating its potential as a viable bioactive molecule. Regarding absorption, **L3** showcases moderate aqueous solubility (logS = −2.418) and a low logP of 0.680, indicating a good balance between hydrophilicity and lipophilicity. It possesses a pKa of 3.343 and an acidic/basic pKa range of 6.170 and 1.360, respectively, indicating that it could exist in both ionized and non-ionized forms at physiological pH, which may enhance absorption. The high Caco-2 permeability (0.942 and −4.144) in different models), human intestinal absorption (HIA) at 0.981, and robust MDCK permeability (0.889) illustrate excellent membrane permeability and intestinal uptake. The high oral bioavailability estimates (F50%, F30%, F20%) spanning from 0.962 to 0.986 further validates its strong absorption profile.

With regards to distribution, the **L3** shows potential BBB permeability (0.967), indicating CNS exposure. It works as an inhibitor of many uptake transporters, including OATP1B1 (0.991) and OATP1B3 (0.996), as well as less for OCT1 and OCT2, which could affect hepatic uptake or drug–drug interactions. It has quite low substrate/inhibitory potential for efflux transporters including BSEP, BCRP, and P-glycoprotein (Pgp), therefore suggesting little threat of resistance mediated by transporters. Its plasma protein binding (PPB) is rather low (0.412), and its volume of distribution (VDss = 0.208) showed moderate tissue permeability.

Regarding metabolism, **L3** serves as a substrate for many cytochrome P450 enzymes (especially CYP1A2, CYP3A4, CYP2B6, CYP2C9, and CYP2C19), indicating metabolic processing along usual hepatic routes. CYP3A4 and CYP2D6, two important enzymes, have little inhibition. Therefore, lowering the chance of major metabolic drug interactions. Strong inhibition is seen as CYP1A2 (0.786). This indicates a moderate metabolic half-life since it exhibits low human and rat liver microsomal stability (HLM = 0.118, RLM = 0.268). Its low UGT substrate potential (0.154) further showed little glucuronidation. In terms of excretion, its predicted plasma clearance (CLp = 0.787) is relatively high, while renal clearance (CLr = 0.267) is moderate, implying a combination of renal and hepatic elimination routes. However, values for half-life (T_1/2_ = −0.434) and mean residence time (MRT = −0.399) suggest a relatively short systemic duration.

Toxicological profiling gives mixed results. Although neurotoxicity is minimal (−2.844), the compound (**L3**) has potential for drug-induced liver injury (DILI = 0.930) and Ames’s mutagenicity rating (0.934). Therefore, implying possible genotoxicity; respiratory (0.312) and reproductive (0.568) risks are moderate. Though cardiotoxicity from hERG inhibition is minor at therapeutic levels (e.g., hERG 1 μM = 0.006), care should still be advised at increased amounts. It shows modestly carcinogenic activity in mouse and rat models as well as possible mitochondrial toxicity (0.691).

Environmental toxicity indicates a high ecological impact, especially on marine life including fish, algae, and *Daphnia magna*. However, the bioconcentration factor (BCF = 0.012–0.445) is low, suggesting a low possibility for bioaccumulation. From a cosmetic and environmental safety standpoint, **L3** has low to medium eye and skin irritation risks, with the potential for phototoxicity (0.681) and photo-allergy (0.845).

Consequently, **L3** showed significant oral absorption, moderate distribution, predictable metabolic routes, and an acceptable excretion profile, although some toxicological factors especially hepatotoxicity, genotoxicity, and photo-reactivity might need additional exploration during lead optimization and preclinical investigations (Table 3, Table 4 and Table 5).

#### 2.3.3. Toxicity Profile and Cosmetic Risk Assessment

The cosmetic risk evaluation of **L3** indicates a combined safety profile concerning topical application and dermal exposure. The compound has a low potential for causing eye corrosion (0.735), yet it has a high probability of inducing eye irritation (0.985), suggesting that direct contact with the eyes could cause considerable discomfort or adverse reactions. Compound **L3** showed a low risk for skin corrosion (0.277) and a moderate potential for skin irritation (0.618), implying it is not excessively corrosive but may still elicit mild to moderate irritation in susceptible individuals. The skin sensitization score of 0.568 reflects a moderate risk of allergic reactions.

In terms of dermal toxicity, the compound presents a relatively high acute dermal toxicity score (0.786), which indicates that absorption through the skin may lead to systemic toxicity at higher doses or concentrations. Additionally, the compound shows significant photo-related risks, with photoinduced toxicity at 0.876, phototoxicity at 0.681, and photoallergy at 0.845. These values imply that exposure to sunlight or UV light alongside the compound could result in adverse effects such as skin inflammation, irritation, or allergic responses. Additionally, although **L3** does not present severe corrosive hazards to skin or eyes, its high potential for irritation, dermal toxicity, and photo-related reactions categorizes it as a candidate requiring careful formulation and safety assessment prior to use in cosmetic products (Table 4 and Table 5).

### 2.4. Brain or Intestinal Estimated (BOILED–Egg) Analysis

**Figure 3:**
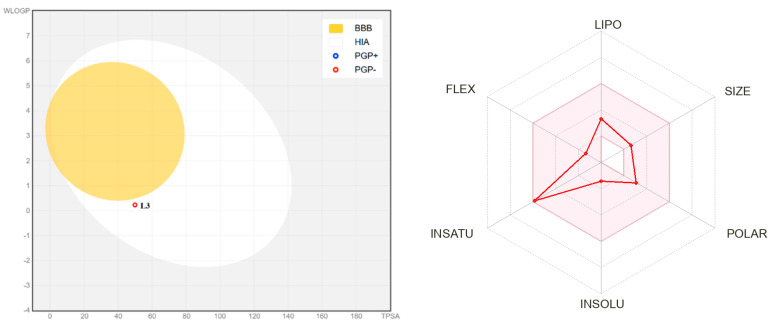
BOILED–Egg plot of the epoxy–functionalized isatin derivative (**L3**).

The WLOGP vs. TPSA plot depicts the estimated HIA and BBB penetration capability of the **L3**. In this plot, the yellow area signifies the ideal zone for BBB permeability, whereas the white zone represents the area for HIA. The red dot, indicating the position of **L3**, is situated within the white HIA region and close to the boundary of the yellow BBB region. This location implies that **L3** is probably well-absorbed in the HIA, suggesting high oral bioavailability. Furthermore, its closeness to the BBB-permeable area signifies that it possesses a moderate to good potential for crossing the BBB, positioning it as a candidate for CNS activity. Furthermore, **L3** exhibits favorable pharmacokinetic characteristics for both systemic and potential CNS-related therapeutic uses. This visualization reinforces prior predictions about the potential oral bioavailability of **L3** and its possible CNS activity, establishing it as a promising candidate for both systemic and potentially CNS-related therapeutic applications (Figure 3).

### 2.5. Molecular Docking Analysis

Protein sequences of *K. pneumoniae* containing the Dam domain were retrieved using UniProt. Despite the absence of a known 3D structure suitable for this protein, a 3D model was successfully prepared using the SWISS–MODEL service (https://swissmodel.expasy.org/ (accessed on 17 May 2025)). The structural feature of the Dam-containing protein in *Escherichia coli* (PDB ID: 4RTJ) was employed as a template for the modelling process. The accuracy and quality of the developed structure were assessed using various computational tools and web servers, including PDBsum and the SAVES server. PROCHECK analysis showed that 89.23% of the phi/psi angle of the parts in the simulated structure was in the best areas, which means the structure is of high quality. Additionally, the ERRAT graph yielded a quality factor of 97.09, signifying potential model reliability, as scores above 50 are generally considered acceptable. We performed further verification using the VERIFY3D server, which confirmed the quality of the model. The model also exhibited a normalized QMEAN4 score within the expected parameters. VERIFY3D analysis demonstrated that 89.23% of residues attained an average 3D-1D score of ≥0.1, thereby validating the structure with a “Pass” outcome.

In this study, docking analysis was performed using two widely recognized tools: AutoDock (AD4) and Vina. As shown in Table 6, the binding energy of the 3D model protein was minimal, indicating a potential binding affinity between **L3** and the protein. The obtained binding energies were −4.29, −4.85, −4.51, and −4.51 kcal/mol, with corresponding inhibition constants of 712.71, 280.35, 494.15, and 494.15 µM. **L3** was positioned within 5.0 Å of the deep active region of the receptor protein, reinforcing its potential interaction with the target site.

A blind docking study further validated the interaction of **L3** with the Dam protein of *K. pneumoniae*. To refine the binding interaction analysis, multiple grids docking (MGD) was employed using AD4, a widely recognized docking software that offers various configuration and docking pathways. The MGD analysis was conducted with a population size of 750, a maximum of 27,000,000 evaluations, and 270,000 generations. To ensure comprehensive analysis, multiple grids with varying dimensions and coordinates were established, covering all identified amino acid residues within the active pocket [58,59,60].

**Figure 4:**
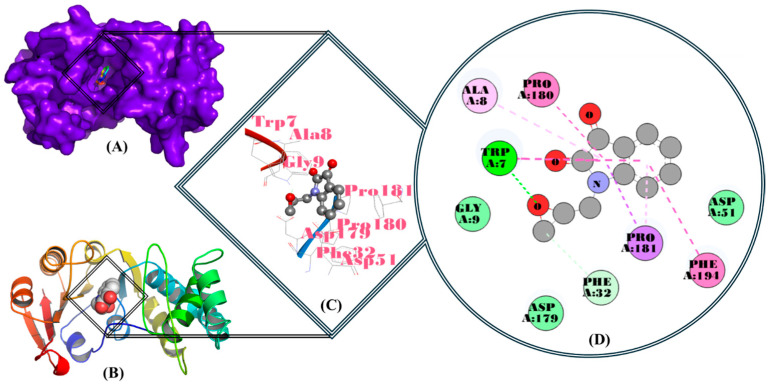
Schematic representations of protein–ligand interactions in the molecular docking of the epoxy-functionalized isatin derivative (**L3**) with the Dam protein of *K. pneumoniae*: (**A**) 3D surface model of the protein with a ball-and-stick representation of the ligand, (**B**) 3D ribbon model of the protein with a CPK representation of the ligand, (**C**) 3D interaction profile illustrating key binding interactions, and (**D**) 2D interaction profile highlighting ligand–protein interactions.

**Figure 5:**
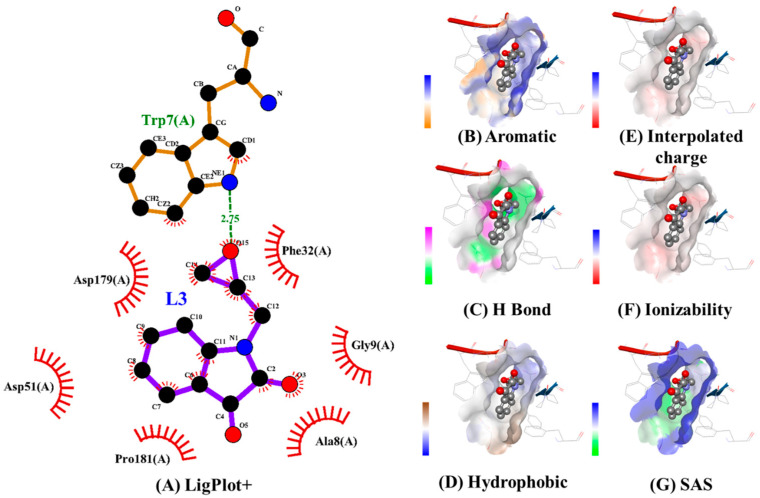
Schematic representation of protein–ligand interactions in the molecular docking study of the epoxy-functionalized isatin derivative (**L3**) with the Dam protein of *K. pneumoniae*: (**A**) 2D LigPlot+ binding pose illustrating key interaction sites, (**B**) visualization of aromatic interactions, (**C**) depiction of hydrogen bond interactions, (**D**) hydrophobicity analysis, (**E**) interpolated charge distribution, (**F**) ionizability assessment, and (**G**) solvent-accessible surface (SAS) representation.

The study identified the optimal grid for the Dam protein, which was determined to be the most favorable pocket for binding interaction analysis. The optimal grid configuration for the Dam protein was set at 30 × 3 Å along the x, y, and z axes, with grid centers positioned at −5.09 Å, 0.63 Å, and 92.86 Å, respectively. Additionally, an alternate grid configuration for the Dam protein was established with dimensions of 56 × 64 × 56 Å, maintaining the same grid center coordinates of −5.09 Å, 0.63 Å, and 92.86 Å (Table 6, Figure 4 and Figure 5).

This study conducted target docking analyses to identify the optimal binding mode and the most suitable grid configuration. The docking tools AD4 and Vina were employed to assess the binding potential of **L3** with the Dam protein. Reports in the literature on Dam inhibitors (such as S-adenosylhomocysteine analogs or DNA-mimicking molecules) having docking scores between −7 and −10 kcal/mol, which suggests comparable binding affinities as **L3** [61,62,63,64]. However, the results demonstrated that **L3** exhibited probable binding capacity and interaction with the target protein. Binding energy calculations using Vina indicated a value of −6.4 kcal/mol, suggesting comparable binding interactions with the available literature. Further analysis revealed that **L3** formed hydrogen bonds with TRP7 and PHE32, while also engaging in van der Waals’ interactions with GLY9, ASP51, and ASP179. These interactions underscore the compound’s potential for effective protein binding (Table 6; Figure 4 and Figure 5).

### 2.6. In Vitro Antibacterial Activity

As shown in Table 7, compound **L3** was active against *B. pumilus*, *B. cereus*, *E. coli*, and *K. pneumoniae*. However, according to their growth inhibition with respect to control, it has been found that **L3** showed the lowest MIC value against *K. pneumoniae* using gentamycin and DMSO as positive and negative controls, respectively.

In this study, compound **L3** showed more significant activity against *K. pneumoniae* (NCTC418) at 93.75 µg/mL. The observed antibacterial activities with other strains were *B. cereus* (MTCC1305), *E. coli* (ATCC 25923), and *B. pumilus* (MTCC 160) at 156.25 μg/mL, 137.25 μg/mL, and 156.25 μg/mL, respectively (Table 7). Thus, the epoxy-functionalized isatin derivative (**L3**) showed the potential MIC activity against *K. pneumoniae* (NCTC418) at 93.75 μg/mL.

## 3. Discussion

**Figure 6:**
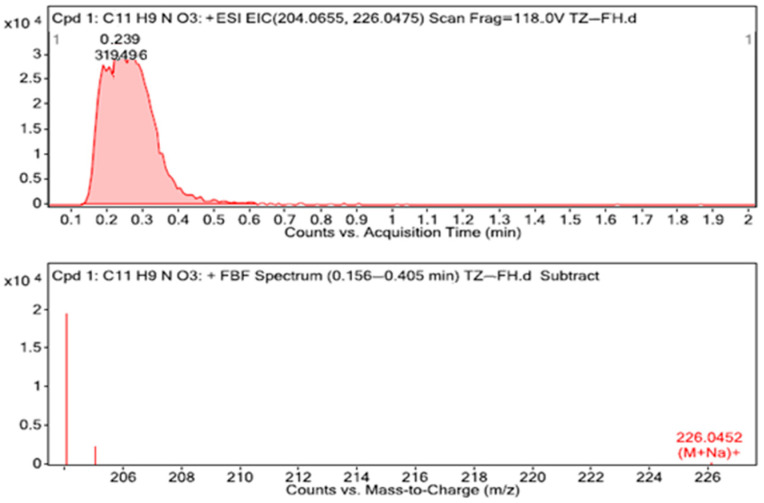
HR–MS plots of the epoxy-functionalized isatin derivative (**L3**).

The UV–Vis spectrum of the synthesized compound (**L3**) displayed an absorption maximum (λ_max_) at 403 nm, measured in an aqueous solution. Even if epoxy groups (oxirane rings) themselves lack powerful chromophores that absorb in the visible spectrum, they could modify the electrical surroundings of nearby conjugated systems. However, when it gets attached to aromatic systems like isatin, the epoxy group might indirectly affect the absorption by means of inductive or mesomeric effects modifying the electron distribution. In the far-UV region (below 220 nm), generally weak n → σ* transitions are normally seen in epoxy groups. However, bathochromic shifts (red shifts) can arise when the epoxy ring is conjugated or close to an extended π-system (such as carbonyl group or aromatic ring), which results in absorbance at longer wavelengths (~ 300–400 nm). As a result, the absorption at 403 nm could result from a lot of π → π* transitions from conjugated aromatic or carbonyl systems, perhaps under the influence of the epoxy ring (Figure 2a). Recorded with KBr pellets, the FT-IR spectrum of the **L3** revealed typical absorption bands at 1194 cm^−1^, which correspond to the C–O–C stretching vibration of the epoxide group, and at 1328 cm^−1^, which is attributed to the C–N stretching related with the epoxide ring. The C–H bending of the epoxide was assigned a band noted at 880 cm^−1^. The stretching vibration of aliphatic N–CH_2_, which connected epoxide group to isatin moiety, was suggested by the absorption at 2888 cm^−1^. Additionally, two distinct carbonyl absorptions at 1727 cm^−1^ and 1614 cm^−1^ on the spectrum relate with C=O stretching vibrations, therefore suggesting the presence of two distinct carbonyl environments in the compound (Figure 2b). The *^1^H*-NMR tests show that protons of the epoxy group showed chemical shifts at 3.4, 3.6, and 3.7–3.8 ppm whereas aromatic protons of isatin moiety showed chemical shifts from 7.1 to 7.6 ppm (Figure 2c). The *^13^C*-NMR spectrum of 1-(oxiran-2-ylmethyl)indoline-2,3-dione was recorded in DMSO-d_6_ at 75 MHz. It shows distinct chemical shifts consistent with the proposed molecular structure. The chemical shift at δ 65.2 ppm is linked to the methylene carbon (–CH_2_–) inside the oxirane ring, whereas the peak at δ 30.8 ppm is related to the methylene carbon (–CH_2_–) linking the oxirane ring to the nitrogen atom of the isatin. Signals observed from aromatic carbons between δ 111.3 and 137.9 ppm suggest different electronic environments of the benzene ring carbons. The peak at δ 151.3 ppm is assigned to the quaternary carbon connected to the nitrogen and conjugated to the carbonyl group. The more downfield peak at δ 65.2 ppm is designated for the oxirane methylene carbon, affected by the electronegative oxygen in the strained three-membered ring. Corresponding to the C-3 and C-2 positions of the isatin ring system, respectively, the two notably deshielded carbonyl carbons are clear at δ 158.6 ppm and δ 183.4 ppm. The observed chemical shifts closely match the structural features of the compound and confirm the presence of both the isatin framework with the epoxide group (Figure 2d). **L3** showed a [M + H]^+^ peak value at 204.06 against the calculated value, which confirms the mass of the tested product (Figure 6).

The epoxy-functionalized isatin derivative (**L3**) shows significant oral absorption, moderate distribution, predictable metabolic routes, and an acceptable excretion profile, although some toxicological factors especially hepatotoxicity, genotoxicity, and photo-reactivity might need additional exploration during lead optimization and preclinical investigations. Furthermore, the **L3** exhibits favorable pharmacokinetic characteristics for both systemic and potential CNS-related therapeutic uses. This visualization reinforces prior predictions about **L3**’s potential oral bioavailability and its possible CNS activity, establishing it as a promising candidate for both systemic and potentially CNS-related therapeutic applications. This study conducted target docking analyses to identify the optimal binding mode and the most suitable grid configuration. The docking tools AD4 and Vina were employed to assess the binding potential of the **L3** with the Dam protein. The results demonstrated that **L3** exhibited the potential binding capacity and interaction with the target protein. Binding energy calculations using Vina indicated a value of −6.4 kcal/mol, suggesting potential binding interactions. Further analysis revealed that **L3** formed hydrogen bonds with TRP7 and PHE32, while also engaging in Van der Waals’ interactions with GLY9, ASP51, and ASP179. 

In this study, **L3** showed significant antibacterial activity against *K. pneumoniae* (NCTC418) at 93.75 µg/mL. Thus, the antibacterial and computational study results showed that the synthesized compound is suitable for further biological evaluation. The docking scores for **L3** as seen in the MIC data reveal that it has affinity for the Dam protein of *K. pneumoniae*, which might explain its biological activity mechanism. It is lower still than the reported binding affinities of well-known or strong Dam inhibitors.

## 4. Materials and Methods

All solvents and reagents utilized in this study were sourced from Sigma-Aldrich, USA, and were of superior quality. They did not undergo any further purification procedures. TLC was conducted on silica gel G employing 100–400 mesh silica gel plates (GF254), and visualized spots were observed using iodine vapor. ^1^H-NMR (400 MHz) was acquired with a Bruker Avance spectrometer (Billerica, MA, USA). DMSO-d_6_ was utilized as the solvent, whereas tetramethylsilane (TMS) used as the internal standard for chemical shifts (δ, ppm). Functional groups were identified using a Bruker vector 22 spectrometer, which operates in the range of 400–4000 cm^−1^, and the identification was performed using potassium bromide pellets or liquid films. Using an Agilent 6200 Series TOF/6500 Series Q-TOF LC/MS system (Santa Clara, CA, USA) equipped with MassHunter Workstation Software version B.05.00 (B5042.0), electro-spray ionization (ESI) in positive mode produced mass spectra.

### 4.1. Method for Synthesis of the Epoxy-Functionalized Isatin Derivative (***L3***)

**Scheme 2:**
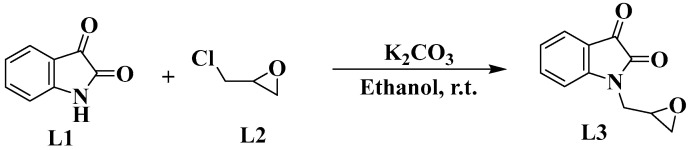
Synthesis of the epoxy-functionalized isatin derivative (**L3**).

In a dry round bottom flask, K_2_CO_3_ (0.5 mmol, 0.069 g) was added to minimum amount of ethanol and stirred for 30 min to one hour. Then, isatin (1 m.mol, 0.147 g) was dissolved for about 15 min. Then, epichlorohydrin (1 m.mol, 77.80 μg/mL) was added, and the mixture was stirred at room temperature for 3–4 h. The reaction was completed after continuously monitoring with TLC (n-hexane:ethyl acetate = 7:3). After completion of reaction, the solvent was removed under reduced pressure. Brown color powdered precipitate was formed. The obtained residue was washed with ethanol, filtered, and dried at room temperature. The precipitate was dissolved in excess amount of ethyl acetate for about 30 min. The mixture was filtered, and the filtrate was collected and dried at room temperature. Powdered precipitate of the residue was collected. Then, the residue obtained was purified using column chromatography, resulting in the desired product **L3** as an orange solid with a yield of 78% (Scheme 2) [34,35].

### 4.2. In Silico Methodology

#### 4.2.1. Drug-Likeness and ADMET Parameters

Using several parameters such as absorption, distribution, metabolism, excretion, and toxicity profiles, *in silico* evaluations of **L3** were carried out with the freely available web tool ADMETlab3 (https://github.com/drfperez/DeepPurpose/blob/main/ADMETLab3.py) and AdmetSAR3 (https://lmmd.ecust.edu.cn/admetsar3/predict.php (accessed on 17 May 2025 and 6 May 2025)) [65,66,67,68]. The physicochemical and ADMET qualities of **L3** validate the drug-like behavior of the compound. In this study, the BOILED-Egg plot was investigated using SwissADME (http://www.swissadme.ch (accessed on 17 May 2025)) [69,70,71,72].

#### 4.2.2. Sequence Retrieval and Homology Modelling

**Figure 7:**
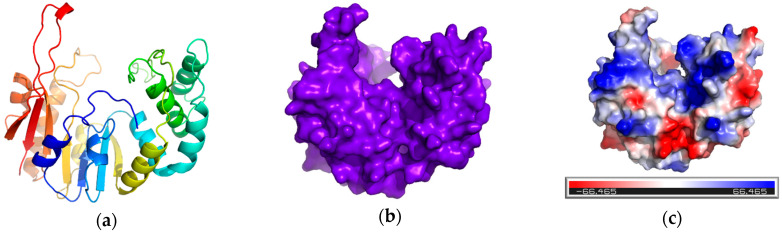
3D structure of modelled protein: (**a**) ribbon, (**b**) surface, and (**c**) electrostatic potential.

The protein sequence of *K. pneumoniae* containing the specified protein domain (UniProt ID: A0A2U0NNR3_KLEPN) was identified using the UniProt database (UniProt link: https://www.uniprot.org). The three-dimensional structure of the target protein was generated using the SWISS-MODEL server (SWISS-MODEL link: https://swissmodel.expasy.org) using fully automated processes [73,74,75,76]. Initially, the structure was constructed using homology modelling and subsequently assessed against a suitable template protein. The template selection was predicated on structural characteristics, with *Escherichia coli* (PDB ID: 4RTJ) designated as the reference (PDB link: https://www.rcsb.org/structure/4RTJ (accessed on 17 May 2025)) [77,78] (Figure 7).

#### 4.2.3. Validation of Modelled Structure

**Figure 8:**
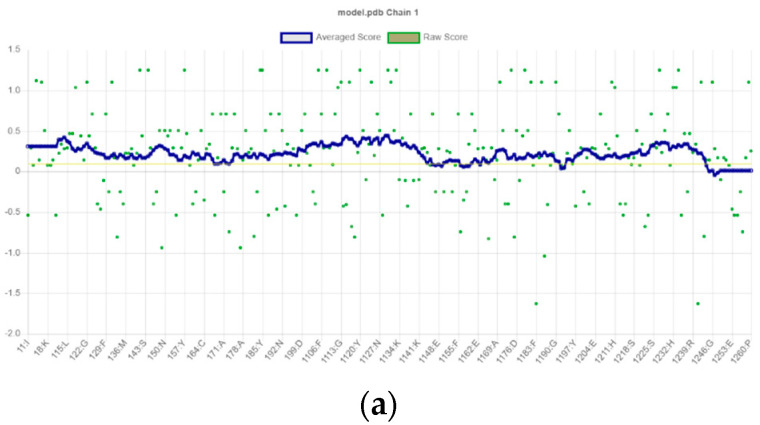

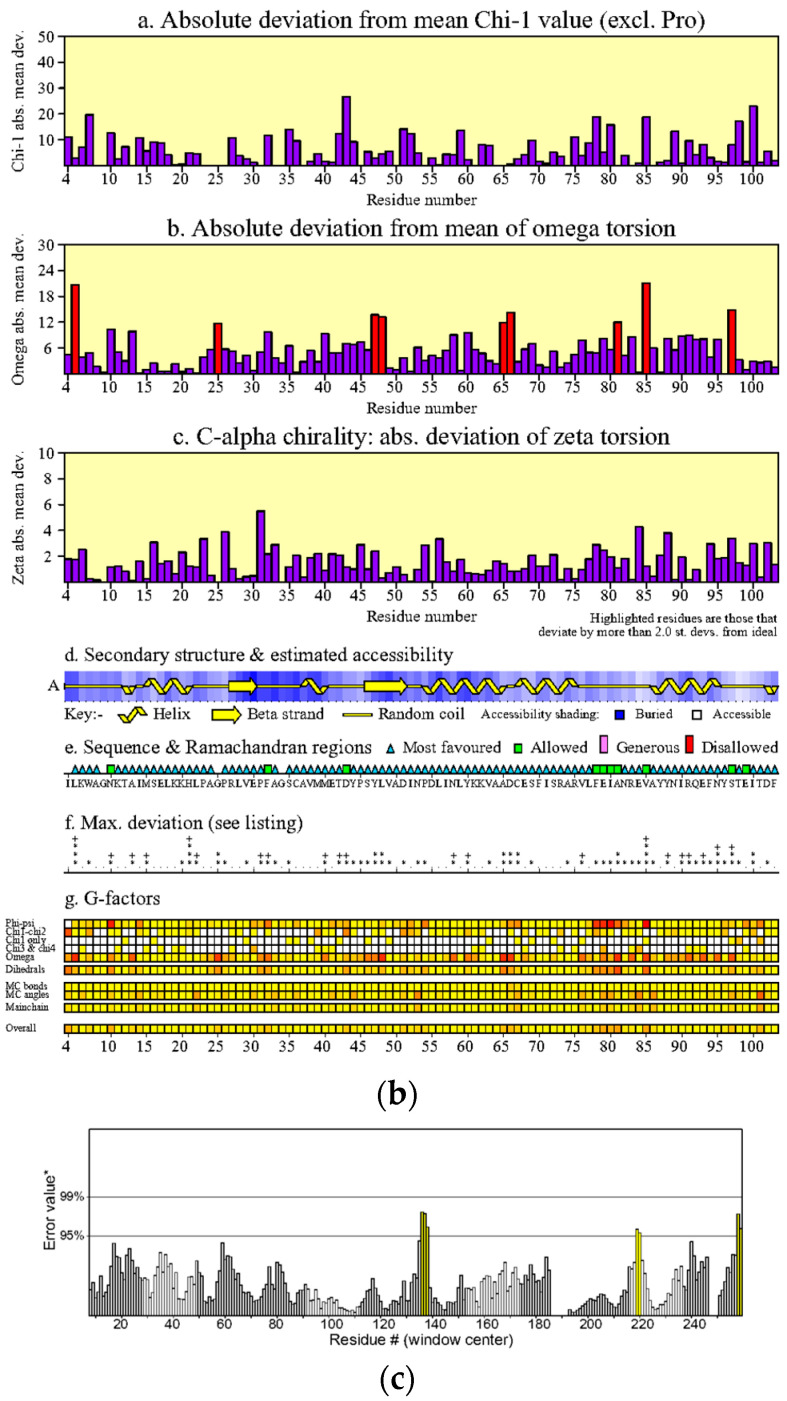
Validation metrics of the 3D structure of the modelled protein. (**a**) The VERIFY3D assessment validates the model’s reliability. (**b**) Residue properties (Max. deviation: the compact histogram of asterisks and plus signs illustrates the maximum deviation of each residue from the ideal values specified in the residue-by-residue). (**c**) ERRAT analysis elucidates the overall quality of the protein structure.

The validation of the model structure was initiated through energy minimization, ensuring its stability and reliability. The optimized structure was then uploaded to the SAVES server, where its accuracy was assessed using ERRAT, and its stereochemical quality was evaluated with PROCHECK [79,80,81,82]. Additionally, Verify3D was employed to examine the compatibility between the atomic model and its corresponding amino acid sequence, further verifying structural integrity [83,84] (Figure 8).

**Figure 9:**
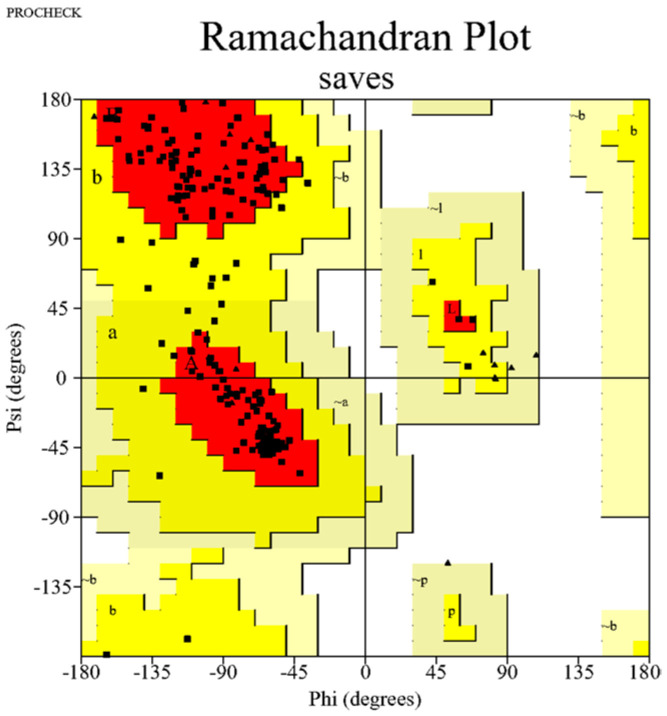
Ramachandran plot of the modelled structure (The plot shows the allowed phi (φ) and psi (ψ) angles for amino acids, which are used to validate the structural correctness of a protein model. Residues in good areas (red/yellow) make sure that things stay stable, while those in acceptable areas (yellow) stay steady but are a little less so. Light yellow residues could mean that there are problems with the structure, while white areas that are not allowed could mean that there are large differences, which could point out errors in the model.).

The PDBsum tool (https://www.ebi.ac.uk/thornton-srv/databases/cgi-bin/pdbsum/ (accessed on 17 May 2025)) was employed to generate the Ramachandran plot and determine the secondary structure of the model protein [85]. This comprehensive analysis was carried out to ensure the accuracy and reliability of the modelled structures, providing critical insights into their conformational integrity (Figure 9).

#### 4.2.4. Active Site Prediction

Active pocket analysis was conducted using FPocketWeb server, providing key insights such as pocket score, druggability, alpha spheres, volume, total SASA, polar SASA, and alpha SASA [86]. To optimize the three-dimensional structure of the target proteins, Swiss-PdbViewer (Swiss-PdbViewer link: https://spdbv.unil.ch/) was utilized. Additionally, non-polar hydrogen, polar hydrogen bonds, and Kollman charges were incorporated into the target proteins using MGL Tools 1.5.7 [87,88,89]. The final structural visualization was carried out with DSV v24.1, ensuring accurate representation and analysis of the 3D models (Table 8).

### 4.3. Antibacterial Method

The antimicrobial assay was performed at the Department of Bioengineering at Integral University, Lucknow, India to examine the antimicrobial potential of the epoxy-functionalized isatin derivative (**L3**).

#### 4.3.1. Determination of the Minimum Inhibitory Concentration

The antimicrobial potential of **L3** was assessed using the standard agar disc diffusion method and MIC according to the Clinical and Laboratory Standards Institute (CLSI, 2015) against the microorganisms tested. The disk diffusion technique was employed for the initial antibacterial assessment of **L3** [90,91,92]. Briefly, *Bacillus pumilus* (MTCC 160), *Bacillus cereus* (MTCC1305), *Escherichia coli* (ATCC 25923), and *K. pneumoniae* (NCTC418) were sourced from NCIM Pune, India. These strains were cultured to the mid-logarithmic phase, collected through centrifugation, rinsed with 10 mM sodium phosphate buffer (SPB) at pH 7.4, and diluted to 2 × 10^5^ colony-forming units (CFU) per mL in SPB supplemented with 0.03% nutrient broth (NB) [93,94]. The **L3** was diluted serially in 100 μL of nutrient broth (NB) medium within 96-well microtiter plates to obtain the target concentrations (62.50–250 μg/mL) alongside a bacterial inoculum (5 × 10^4^ CFU per well). Following an overnight incubation at 37 °C, the MIC was defined as the lowest concentration of the **L3** at which bacterial growth was inhibited.

MIC = (Lowest concentration inhibit growth + Highest concentration allow growth)/2

#### 4.3.2. Control

Gentamycin served as the standard antibacterial control, while DMSO used as the negative control for the purpose of comparing results under the same conditions. Following the determination of the MIC of bacteria from the wells of the microtiter plate showing no visible growth, samples were taken for serial subculture of 2 μL into microtiter plates containing 100 μL of broth per well and incubated further for 24 and 48 h. The lowest concentration that exhibited no visible growth was identified as MBC, signifying 99.97% elimination of the original inoculum. The optical density of each well was assessed at a wavelength of 595 nm using an ELISA Reader (BORAD United States of America) and was compared against a blank [95,96]. Two replicates were conducted for compound **L3**, and each experiment was repeated three times.

## 5. Conclusions

This study successfully synthesized a epoxy-functionalized isatin derivative (**L3**) using a one-pot reaction. Comprehensive characterization *via* FT-IR, *¹H*-NMR, HR-MS, and UV–Vis spectroscopy confirmed its structural integrity. The antibacterial potential of **L3** was tested using MIC assays on *B. cereus*, *B. pumilus*, *E. coli*, and *K. pneumoniae*, showing that it was most effective against *K. pneumoniae* with a MIC value of 93.75 μg/mL. *In silico* evaluations using ADMETlab3 and SwissADME indicated favorable drug-like properties, including potential oral bioavailability, BBB permeability, and low PPB. The compound met Lipinski’s and other drug-likeness rules, achieving a QED score of 0.52. Molecular docking studies found that DNA adenine methyltransferase (Dam) could be an effective target for antibiotics studies in *K. pneumoniae*. Since, a model was developed using the SWISS-MODEL server and checked their accuracy with the help of *in silico* tools. Docking studies showed that **L3** binds well to Dam, with binding energies of −6.4 kcal/mol (Vina) and −4.85 kcal/mol (AutoDock) and an inhibition constant of 280.35 µM. Further analysis showed that **L3** forms hydrogen bonds with TRP7 and PHE32 and has Van der Waals’ interactions with GLY9, ASP51, and ASP179. In addition, the ADMET profile, physical, and chemical characteristics of **L3** correlate with the known drug-like standards, showing that it is a considerable candidate for more research. These results highlight **L3** as a possible antibacterial candidate that could be used for developing alternative antimicrobial candidate, especially aimed to target the Dam protein in *K. pneumoniae* to control *K. pneumoniae*-mediated bacterial infection. Therefore, **L3** presents a promising basis as a unique scaffold, yet further structural optimization is necessary to enhance its binding affinity and overall efficacy. Incorporating comparative *in vitro* and *in vivo* analysis in future studies could further validate its potential as a targeted antibacterial agent.

## Figures and Tables

**Table 1:** Physiochemical properties of the epoxy-functionalized isatin derivative (**L3**).

Physicochemical Property		
**Parameters**	**L3**	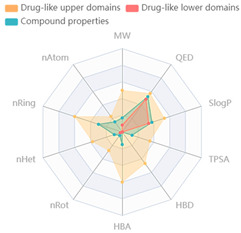
Molecular Weight (MW)	203.197	
Number of atoms (nAtom)	15	
Number of heteroatoms (nHet)	4	
Number of rings (nRing)	3	
Number of rotatable bonds (nRot)	2	
Hydrogen bond acceptors (HBA)	3	
Hydrogen bond donors (HBD)	0	
Topological polar surface area (TPSA)	49.91	
Lipophilicity (logP)	0.615	

**Table 2:** Medicinal chemistry characteristics of the epoxy-functionalized isatin derivative (**L3**).

Medicinal Chemistry	
Parameters	L3
Quantitative estimate of drug-likeness (QED)	0.52
Lipinski Rule (Pfizer’s Rule of Five/Rule of Five/RO5)	Accept
GSK Rule (GlaxoSmithKline rule/Veber rule)	Accept

**Table 3:** Absorption profile of the epoxy-functionalized isatin derivative (**L3**).

Absorption	
**Parameters**	**L3**
Logarithm of aqueous solubility (logS)	−2.418
Logarithm of the partition coefficient (logP)	0.680
Acid dissociation constant (Acidic pKa)	6.170
Base dissociation constant (Basic pKa)	1.360
Human colon carcinoma cell line (Caco-2)	0.942
Human Intestinal Absorption (HIA)	0.981
Madin-Darby Canine Kidney cells (MDCK)	0.889
Fraction of drug absorbed at 50% concentration (F50%)	0.962
Distribution	
Blood-Brain Barrier (BBB)	0.967
OATP2B1 inhibitor	0.055
OCT1 inhibitor	0.121
OCT2 inhibitor	0.050
Breast Cancer Resistance Protein (BCRP) inhibitor	0.073
Bile Salt Export Pump (BSEP) inhibitor	0.063
Multidrug And Toxin Extrusion Protein 1 (MATE1) inhibitor	0.051
Permeability Glycoprotein (Pgp) inhibitor	0.031
Permeability Glycoprotein (Pgp) substrate	0.034
Plasma Protein Binding (PPB)	0.412
Metabolism	
CYP1A2 inhibitor	0.786
CYP3A4 inhibitor	0.006
CYP2B6 inhibitor	0.235
CYP2C9 inhibitor	0.131
CYP2C19 inhibitor	0.209
CYP2D6 inhibitor	0.008
CYP1A2 substrate	0.775
CYP3A4 substrate	0.510
CYP2B6 substrate	0.649
CYP2C9 substrate	0.535
CYP2C19 substrate	0.668
CYP2D6 substrate	0.275
Human Liver Microsomes (HLM)	0.118
Rat Liver Microsomes (RLM)	0.268
UDP-Glucuronosyltransferase (UGT) substrate	0.154
Excretion	
Plasma Clearance (CLp)	0.787
Renal Clearance (CLr)	0.267
Half-Life (T1/2)	−0.434
Mean Residence Time (MRT)	−0.399

**Table 4:** Toxicity profile of the epoxy-functionalized isatin derivative (**L3**).

Human Health Toxicity	
**Parameters**	**L3**
Organ toxicity	
Neurotoxicity	−2.844
Drug-Induced Liver Injury (DILI)	0.930
human ether-a-go-go related gene (hERG) 1 µM	0.006
human ether-a-go-go related gene (hERG) 1–10 µM	0.004
Respiratory toxicity	0.312
Nephrotoxicity	0.838
Toxicity endpoint	
Ames’s mutagenesis	0.934
Mouse carcinogenicity	0.775
Rat carcinogenicity	0.794
Rat carcinogenicity	0.932
Rodents’ carcinogenicity	0.884
Micronucleus	0.933
Acute oral toxicity	0.893
Endocrine disruption	
Androgen Receptor (AR)	0.044
Estrogen Receptor (ER)	0.008
Aromatase	0.046
ATPase Family AAA Domain-Containing Protein 5 (ATAD5)	0.019
Heat Shock Element (HSE)	0.037
Tumor Protein p53 (p53)	0.088
Peroxisome Proliferator-Activated Receptor (PPAR)	0.030
Matrix Metalloproteinases (MMP)	0.057
Thyroid Hormone Receptor (TR)	0.023
Glucocorticoid Receptor (GR)	0.058
Ecological Risk Assessment	
Terrestrial organisms	
Honeybee toxicity	0.266
Aquatic organisms	
Fish toxicity	0.492
Fathead minnow toxicity	0.424
Bluegill sunfish toxicity	0.595
Rainbow trout toxicity	0.649
Sheepshead minnow toxicity	0.451
Other	
Bioconcentration Factor (BCF)	0.012
Biodegradability	0.235

**Table 5:** Cosmetic risk assessment of the epoxy-functionalized isatin derivative (**L3**).

Cosmetic Risk Assessment		
**Parameters**	**L3**	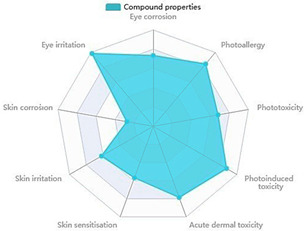
Eye corrosion	0.735	
Eye irritation	0.985	
Skin corrosion	0.277	
Skin irritation	0.618	
Skin sensitization	0.568	
Acute dermal toxicity	0.786	
Photoinduced toxicity	0.876	
Phototoxicity	0.681	
Photoallergy	0.845	

**Table 6:** Molecular docking analysis of the epoxy-functionalized isatin derivative (**L3**) against the Dam protein in *K. pneumoniae*, with the blind and target grids using AutoDock and Vina.

Grid Size (Å)			Grid Coordinates (Å)			BE (kcal/mol)		K_d_ (µM)	Most Interacting Amino Acids		
X	Y	Z	X	Y	Z	AD4	Vina		VDW	H-Bond	π-π
56	64	56	−5.09	0.63	92.86	−4.29	−6.4	712.71	TRP7, ALA8, GLY9, PHE32, GLY34, ASP179, TYR182, PHE194	-	PRO181
30	30	30	−5.09	0.63	92.86	−4.85	−6.4	280.35	GLY9, ASP51, ASP179	TRP7, PHE32	ALA8, PRO180, PRO181, PHE194
30	30	30	−2.79	0.63	94.50	−4.51	−6.4	494.15	PHE32, ASP179, PRO180, TYR182	TRP7, GLY9	ALA8, PRO181
30	30	30	−4.99	−1.45	95.57	−4.51	−6.4	494.15	PHE32, ASP179, PRO180, TYR182	TRP7, GLY9	ALA8, PRO181

**Table 7:** Antibacterial activity (MIC μg/mL) of the epoxy-functionalized isatin derivative (**L3**).

Microbial Strains	MIC Value of L3	MIC of Gentamycin	SD	ER. BAR
*B. cereus*	156.250	80	±0.519	0.416
*B. pumilus*	156.250	80	±0.527	0.419
*K. pneumoniae*	93.750	80	±0.650	0.465
*E. coli*	137.500	80	±0.548	0.427

**Table 8:** Detected top three pockets of protein as active sites.

**Pocket**	**Score**	**Draggability**	**Alpha Spheres**	**Volume**	**Total SASA**	**Polar SASA**	**Alpha SASA**	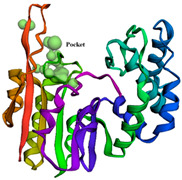
I	0.985	0.985	117	1293	123.153	85.717	37.436	
II	0.554	0.036	48	443.142	45.001	45.001	0.000	
III	0.511	0.064	42	447.774	28.403	23.572	4.831	

## Data Availability

The datasets used and/or analyzed during the current study are available from the corresponding author on reasonable request.
